# Mining key genes related to root morphogenesis through genome-wide identification and expression analysis of *RR* gene family in citrus

**DOI:** 10.3389/fpls.2022.1068961

**Published:** 2022-11-22

**Authors:** Manman Zhang, Fusheng Wang, Xiaoli Wang, Jipeng Feng, Qian Yi, Shiping Zhu, Xiaochun Zhao

**Affiliations:** ^1^ Citrus Research Institute, Southwest University/Chinese Academy of Agricultural Sciences, Chongqing, China; ^2^ National Citrus Engineering Research Center, Chongqing, China

**Keywords:** citrus, root system architecture, response regulators, gene family, abiotic stress

## Abstract

Morphogenesis of root is a vital factor to determine the root system architecture. Cytokinin response regulators (RRs) are the key transcription factors in cytokinin signaling, which play important roles in regulating the root morphogenesis. In this study, 29 RR proteins, including 21 RRs and 8 pseudo RRs, were identified from the genome of citrus, and termed as CcRR1-21 and CcPRR1-8, respectively. Phylogenetic analysis revealed that the 29 CcRRs could be classified into four types according to their representative domains. Analysis of *cis*-elements of *CcRRs* indicated that they were possibly involved in the regulation of growth and abiotic stress resistance in citrus. Within the type A and type B *CcRRs*, *CcRR4*, *CcRR5*, *CcRR6* and *CcRR16* highly expressed in roots and leaves, and dramatically responded to the treatments of hormones and abiotic stresses. *CcRR2*, *CcRR10*, *CcRR14* and *CcRR19* also highly expressed in roots under different treatments. Characteristic analysis revealed that the above 8 *CcRRs* significantly and differentially expressed in the three zones of root, suggesting their functional differences in regulating root growth and development. Further investigation of the 3 highly and differentially expressed *CcRRs*, *CcRR5*, *CcRR10* and *CcRR14*, in 9 citrus rootstocks showed that the expression of *CcRR5*, *CcRR10* and *CcRR14* was significantly correlated to the length of primary root, the number of lateral roots, and both primary root and the number of lateral roots, respectively. Results of this study indicated that *CcRRs* were involved in regulating the growth and development of the root in citrus with different functions among the members.

## Introduction

Root system architecture is a critical characteristic to determine the distribution of roots in soil, the capacity of absorption, anchorage and stress resistance (Lavenus et al., 2013; Singh et al., 2020; Jeon et al., 2021). Formation of root system architecture is a complex biological process regulated by multiple genes, of which the expression greatly affected by environmental conditions. Plant root can sense signals of any environmental changes and modify its primary root length and lateral root density through cell proliferation and cell differentiation to adapt to the changes of environmental factors (Street et al., 2016; Liu and Wirén, 2022). Phytohormones such as auxin, cytokinin, abscisic acid etc., play essential roles in root morphogenesis and growth (de Bang et al., 2020; Pandey et al., 2021; Deng et al., 2022; Ji et al., 2022; Li C. et al., 2022; Timilsina et al., 2022).

Cytokinin, a plant-specific chemical messenger, plays an important role in many aspects of plant growth and development, such as cell division and differentiation of the root meristem, vascular development, stress response and plant immunity (Choi et al., 2010; Ni et al., 2017; Li L. et al., 2022; Papon and Courdavault, 2022). It acts as a potent negative regulator of root formation and elongation, including primary root, lateral root and crown root (Li L. et al., 2022). The cytokinin signaling system is a two-component system, mainly consisting of three important parts: receptor protein histidine kinase (HK), histidine-phosphotransfer protein (HP), and response regulator (RR) (Muller and Sheen, 2007). Among them, RRs play crucial roles in cytokinin signal transduction.

In *Arabidopsis*, the RR family contains 24 members, which can be divided into four types, type A, type B, type C and pseudo ARR (Makino et al., 2000; Horák et al., 2008; Schaller et al., 2008). Type A ARRs have a conserved aspartic acid-aspartic acid-lysine (D-D-K) region and a short C-terminal with unknown functions, and their expression could be induced by exogenous cytokinin, which occurs in the absence of *de novo* protein synthesis (To et al., 2004). In addition to the D-D-K domain, type B ARRs contain a long C-terminal sequence encoding a MYB-like DNA binding domain, called ARRM, which is the binding site to downstream target genes for cytokinin signaling (Ramireddy et al., 2013; Sakai et al., 2001). Type B ARRs serve as transcriptional regulators for certain target genes such as the type A *ARR* genes (Tajima et al., 2004). Type C ARRs have a domain similar to that of the type A ARRs in structure. However, their expression could not be induced by cytokinin (Yang et al., 2016). Pseudo ARRs lack conserved residues for phosphorylation and some of them contain a CCT (for CONSTANS, CONSTANS-like and TOC1) motif in C-termini (Strayer et al., 2000; To et al., 2007; Li et al., 2017).

RR family members were reported to be involved in root development. Previous study showed that ARR1 and ARR12 could bind to the promoter of *LAX2*, an auxin influx carrier, and inhibited its expression, thus regulating the distribution of auxin in the root apical meristem (Zhang et al., 2013). ARR1 mediated arsenate-induced root growth inhibition by directly binding to the promoters of *ASA1* (*ANTHRANILATE SYNTHASE ALPHA SUBUNIT*) and *ASB1* (*BETA SUBUNIT 1*), and positively regulating their expressions (Tu et al., 2021). Similarly, ARR2 and ARR12 could affect the formation of lateral root primordia by directly and negatively regulating the localization of PIN-FORMED (PIN) proteins, the key factors in regulating cell division and differentiation of root meristem by maintaining adequate auxin concentration (Hwang et al., 2012). The up-regulated *ARR16* and *ARR17* at the lower water potential side of root were involved in root hydrotropism through promoting the division of cells in the meristem zone (Chang et al., 2019). *RcRR1*, a *Rosa canina* type A response regulator gene, homology to *ARR8* and *ARR9*, was reported to be involved in cytokinin-modulated rhizoid organogenesis, and overexpression of *RcRR1* resulted in increased primary root length and lateral root density (Gao et al., 2013). Citrus is the most important fruit plant worldwide. However, little attention has been paid to RRs in citrus research. Previous study in our group indicated that *RRs* probably play essential roles in citrus rootstock roots by RNA-seq analysis (unpublished data).

In this study, the *RR* genes were comprehensively identified and characterized in the whole citrus genome. The expression profiles of type A and type B *CcRRs* were analyzed in different plant tissues and organs, under different hormones and stresses treatments as well. Expressions of *CcRRs* in different zones of root and varieties were also analyzed to mine the functional genes related to root morphogenesis in citrus rootstocks. It is a crucial basis for further study on the regulation of *RR* genes in citrus root morphogenesis, and provides guidance for genetic modification of rootstocks to solve the issues faced in citriculture.

## Materials and methods

### Plant materials

The nine citrus rootstock varieties, including *Poncirus trifoliata* (Pt026, Pt030, Pt034, Pt038), *Citrus wilsonii* Tanaka ‘Zhique’, *C. limonia* Pasquale ‘Volkamer’, *C. limonia* Osbeck ‘Canton Lemon’, *C. reshni* Hort. Ex Tan ‘Cleopetra’, and *C. reticulata* ‘Zhuju’, were used in this study. The plant materials were collected from National Citrus Germplasm Repository (Chongqing, China).

The seedlings of ‘Volkamer’ were used for analysis of genes expression in different tissues and organs, and under different hormone treatments and stresses. The seedlings of all varieties were used to study the correlation between root indexes and gene expression.

### Genome-wide identification of *RR* gene family in citrus

The genome and protein information of citrus plant (*C. clementina*) was downloaded from the Phytozome database (http://phytozome.jgi.doe.gov/pz/portal.html). Twenty-four response regulators’ protein sequences of *Arabidopsis thaliana* (http://www.arabidopsis.org/) (ARRs) were used as reference sequences to identify the members of the RR family in *C. clementina* (CcRRs) with protein basic local alignment search tool (BLASTP) (Mahram and Herbordt, 2015). The top E-value was less than 1 × 10^−10^. All the retrieved sequences were submitted to the HMMER database (https://www.ebi.ac.uk/Tools/hmmer/; Potter et al., 2018), Pfam database (http://pfam.xfam.org/; Punta et al., 2011) and NCBI CDD database (https://www.ncbi.nlm.nih.gov/Structure/cdd/wrpsb.cgi; Marchler-Bauer et al., 2015) to confirm whether they were the members of RR family. The redundant members were removed manually. ExPASy website (http://web.expasy.org/) was used to predict molecular weight (MW) and isoelectric points (pI) of all proteins of the identified RR family members. CELLO website (http://cello.life.nctu.edu.tw/) was used to predict the subcellular localization of RR family members (Yu et al., 2006).

### Chromosomal distribution

The chromosomal location of each *RR* gene was derived from the genome annotation information. Chromosomal distributions of these genes were generated using MapChart software (Voorrips, 2002).

### Phylogenetic analysis

MEGA7.0 software (Kumar et al., 2016) was used to construct the phylogenetic tree with protein sequences of RRs from different plant species based on the Maximum Likelihood Method (MJ). The bootstrap value was repeated 1000 times.

### Gene structure, motif and promoter analysis

TBtools (Chen et al., 2020) was used to display the distribution of gene structure of *RRs*. The analysis of the protein conserved motif was conducted through the online MEME website (https://meme-suite.org/meme/tools/meme; Bailey and Elkan, 1995) to study the differences among RR family members. The maximum number of motifs was set to 5. 2000 bp upstream sequence of each *RR* gene was extracted from the citrus genome. The *cis*-elements in the promoter region were predicted using PlantCARE database (http://bioinformatics.psb.ugent.be/webtools/plantcare/html/) (Lescot et al., 2002).

### Treatments

After removal of seed coats, the embryos were cultured in petri dish at 28°C under moisturized conditions for 3-5 days for germination. The seedlings of Volkamer were grown in hydroponics for 25 days. The roots, stems and leaves were collected for gene expression analysis of *CcRRs*. Meanwhile, the remaining seedlings were treated with hormones and abiotic stresses. The root samples were collected at different time (0, 1, 3, 6, 12 h) after 50 μM 6-BA, 100 μM ABA, and 20% polyethylene glycol (PEG_6000_) treatments for RNA extraction, respectively. The roots were also sampled from the seedlings treated at 4°C and 200 mM NaCl for 0, 3, 6, 12 and 24 h, respectively. In addition, the seedlings with lateral root primordium just emerged (as shown in **Supplementary Figure 1**) were used to explore the expression of type A and type B *RRs* in different zones of root (RT: the meristematic/elongation zone; RM: the root elongation/differentiation and lateral root initiation zone; RC: lateral root growth zone). This division was based on the reports of Hwang et al. (2012), Goh (2019), Santos Teixeira and ten Tusscher (2019).

The seeds of nine citrus rootstocks under same state were selected and sown in the pots (30 × 30 cm) filled with the mix of vermiculite and perlite. Different zones of root were sampled at 25, 50 and 75 days after sowing. The samples were immediately frozen with liquid nitrogen and stored at -80°C for RNA extraction to explore the expression of the three *RR* genes. At the same time, the length of primary root and the number of lateral roots in RC zone of the root were counted. All the seedlings were grown at 28°C in a greenhouse under 16 h day length.

### RNA extraction and qRT-PCR

Total RNA was isolated from samples using an EASYspin Plus Plant RNA Rapid Extraction Kit RN38 (Aidlab, Beijing, China) following the manufacturer’s instructions. Subsequently, the RNA was reverse transcribed using the Thermo Scientific RevertAid MM (Thermo Fisher, Lithuania). qRT-PCR was performed with 10 μL reaction mixture containing 2 μL cDNA sample, 5 μL universal SYBR^®^ Green Supermix (2 × iTaq™, Bio-Rad, CA, USA), 0.2 μL of each primer (10 μM) and 2.6 μL sterilized water. The qRT-PCR program was as follows: 95°C for 60 s, followed by 39 cycles of 95°C for 20 s, 60°C for 30s. The primers used in this study are listed in **Supplementary Table 1**. Each treatment included three replications. The expression levels of the genes were normalized with citrus *Actin* gene, and fold changes were calculated using 2^-ΔΔCt^ method (Zhang et al., 2020).

## Results

### Identification of CcRR family members in citrus

In this study, more than 100 RRs and RR like proteins were searched out from the *C. clementina* genome. The RRs with sequence redundancy or alternative splicing forms were removed. Furthermore, the RRs containing other domains, or lacking conserved motifs were also eliminated. Finally, a total of 29 potential RR proteins (including 8 pseudo RRs) were identified, and named as CcRR1-21 and CcPRR1-8 according to their positions on the scaffolds. The details of CcRRs, including their names, accession numbers, number of amino acids, molecular weight, isoelectric point (pI), grand average of hydropathicity (GRAVY) and putative subcellular localization were listed in **Table 1**. The number of amino acids of CcRR proteins varied from 140 (CcRR12) to 873 (CcPRR7), with molecular weight (MW) from 15.29 (CcRR12) to 95.44 kDa (CcPRR7) and pI from 4.88 (CcRR19) to 8.92 (CcRR21). The prediction of subcellular localization showed that most of CcRRs were located in the nucleus, and a few of them were located in the plasma membrane, mitochondria or chloroplast.

**Table 1:** Physicochemical properties and subcellular localization prediction of CcRRs.

Family members	Gene ID	Number of amino acids (aa)	Molecular weight (Da)	Theoretical pI	Grand average of hydropathicity (GRAVY)	Subcellularlocalization
CcRR1	Ciclev10007639m	695	77100.92	6.78	-0.514	Nuclear
CcRR2	Ciclev10007648m	681	74487.07	5.49	-0.527	Nuclear
CcRR3	Ciclev10017880m	145	16181.99	7.83	-0.106	Cytoplasmic
CcRR4	Ciclev10014525m	663	71705.54	5.62	-0.325	Nuclear
CcRR5	Ciclev10022334m	199	21885.21	5.2	-0.106	Chloroplast; Nuclear
CcRR6	Ciclev10021937m	242	27525.66	5.32	-0.918	Nuclear
CcRR7	Ciclev10024122m	141	15852.3	6.71	-0.362	Cytoplasmic; Nuclear; Mitochondrial
CcRR8	Ciclev10032983m	152	16509	5.42	-0.214	Nuclear
CcRR9	Ciclev10033605m	517	56319.71	4.91	-0.368	Nuclear
CcRR10	Ciclev10031052m	584	65413.85	5.76	-0.454	Nuclear
CcRR11	Ciclev10032281m	292	32223.62	5.04	-0.1	Plasma Membrane; Nuclear
CcRR12	Ciclev10003634m	140	15290.59	6.06	-0.024	Cytoplasmic; Nuclear
CcRR13	Ciclev10002312m	241	27334.8	4.93	-0.763	Nuclear
CcRR14	Ciclev10004470m	680	73957.89	5.75	-0.477	Nuclear
CcRR15	Ciclev10005747m	241	27744.13	6.32	-0.334	Cytoplasmic; Nuclear
CcRR16	Ciclev10004999m	436	48778.97	6.95	-0.314	Nuclear
CcRR17	Ciclev10006814m	145	16010.7	5.81	-0.101	Cytoplasmic
CcRR18	Ciclev10006872m	145	16079.85	5.75	-0.045	Cytoplasmic
CcRR19	Ciclev10005987m	187	21058.11	4.88	-0.341	Nuclear
CcRR20	Ciclev10006586m	593	65047.43	6.75	-0.419	Nuclear
CcRR21	Ciclev10006834m	593	64233.92	8.92	-0.46	Nuclear
CcPRR1	Ciclev10019534m	558	62582.02	5.55	-0.708	Nuclear
CcPRR2	Ciclev10019177m	670	74513.11	6.51	-0.682	Nuclear
CcPRR3	Ciclev10023753m	689	75527.12	6.5	-0.613	Nuclear
CcPRR4	Ciclev10019943m	479	53581.39	6.29	-0.331	Nuclear
CcPRR5	Ciclev10030911m	660	71465.42	8.53	-0.744	Nuclear
CcPRR6	Ciclev10031120m	559	62348.41	5.91	-0.627	Nuclear
CcPRR7	Ciclev10027794m	873	95436.77	6.24	-0.803	Nuclear
CcPRR8	Ciclev10011108m	786	85493.04	7.54	-0.726	Nuclear

### Phylogenetic analysis and chromosomal distribution of CcRRs

**Figure 1:**
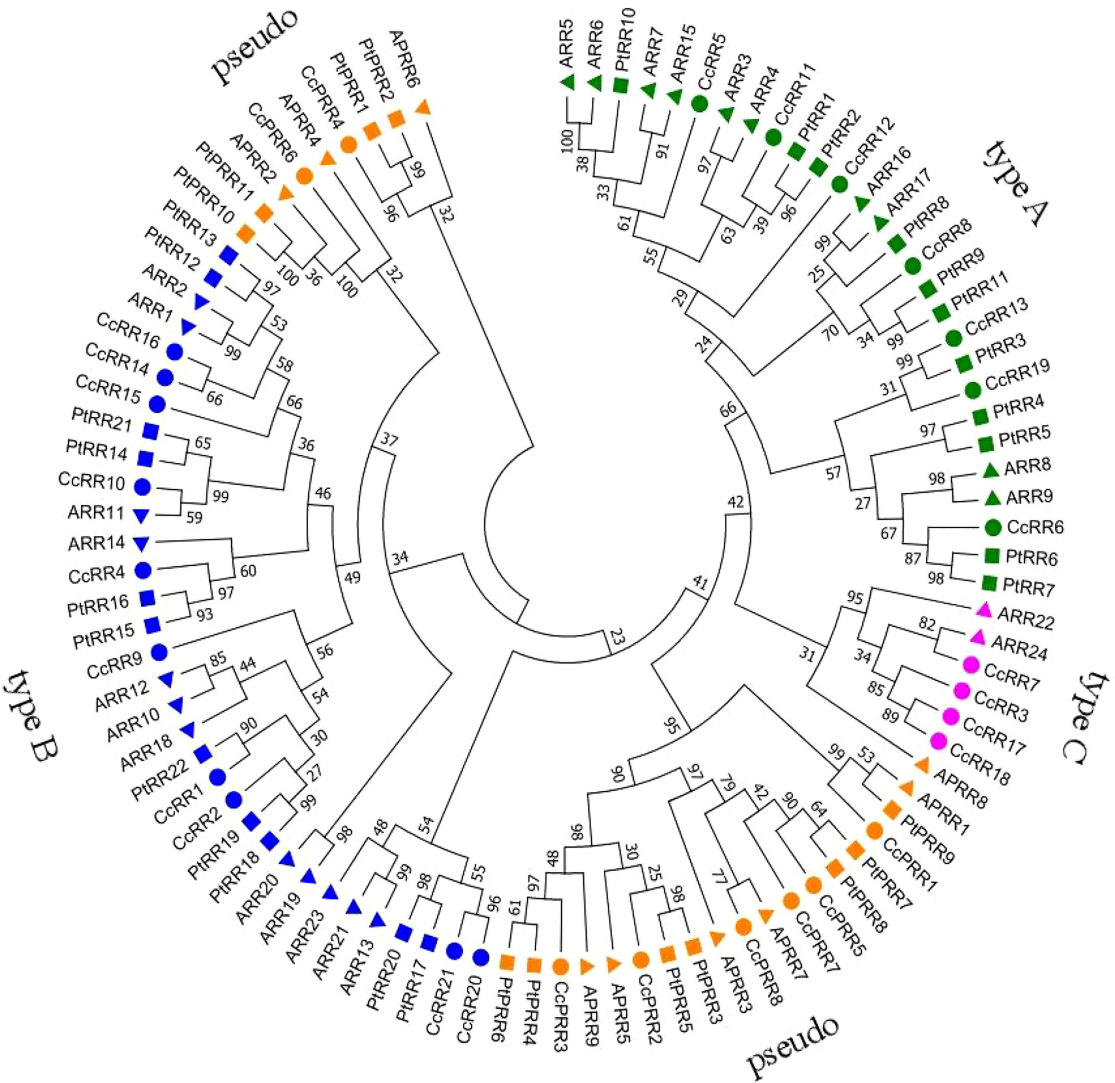
Phylogenetic tree of RR proteins from *Citrus clementina, Arabidopsis thaliana and Populus trichocarpa.* Groups with different colors represent different types of RRs (Green: type A RRs, with only one D-D-K domain; Blue: type B RRs, with D-D-K domain and MYB-like DNA-binding domain; Pink: type C RRs, with a D-D-K domain similar to type-A CcRRs; Orange: pseudo RRs, with D-D-K domain and CCT domain, except for CcPRR4 and CcPRR6).

**Figure 2:**
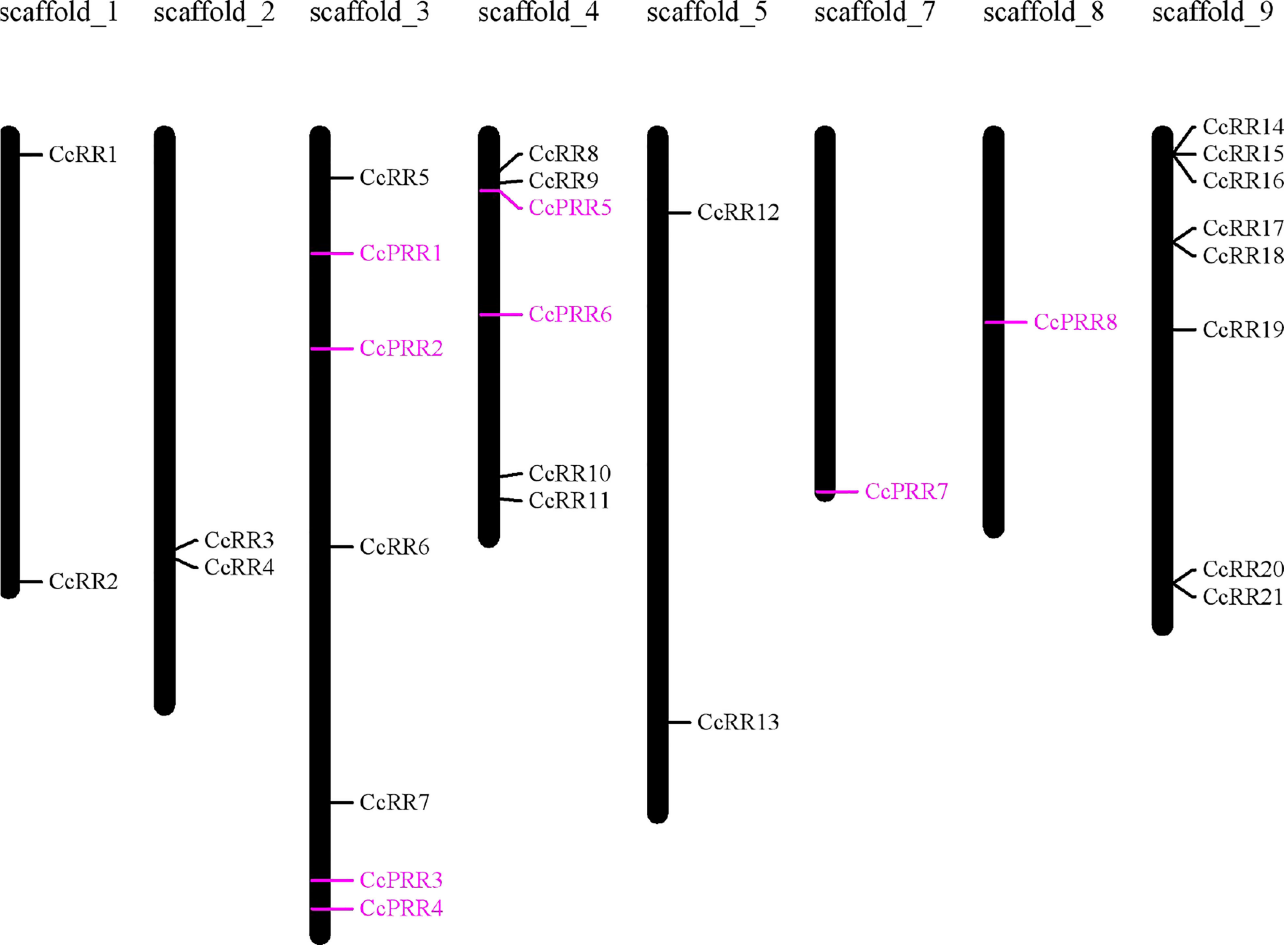
Chromosome location of *CcRR* gene family members on the *C. clementina* scaffolds. Pseudo *RRs* were indicated in purple colour.

To uncover the phylogenetic relationship of CcRRs, a phylogenetic tree was generated with 95 RR proteins, of which 33 were from *Arabidopsis* (24 ARR and 9 APRR), 33 from poplars (22 PtRR and 11 PtPRR) (Ramírez-Carvajal et al., 2008), and 29 from citrus (21 CcRR and 8 CcPRR) (**Figure 1**). It showed that the 95 RRs were divided into 9 clusters. Based on the feature of protein structure, the 9 clusters could be classified into 4 types. The group in green represented type A CcRRs with only one D-D-K domain, including CcRR5-6, CcRR8, CcRR11-13 and CcRR19. Type C CcRR (pink) was mostly close to type A with a similar D-D-K domain. It is a small group with only four members in citrus (CcRR3, CcRR7 and CcRR17-18). CcRR1-2, CcRR4, CcRR9-10, CcRR14-16 and CcRR20-21 were placed in type B CcRRs (blue) with D-D-K domain and MYB-like DNA-binding domain. The group marked in orange color contained pseudo RRs with D-D-K domain and CCT domain. Chromosome mapping results showed that most of CcRR members were clustered on scaffold 3, 4 and 9, and a few of them were on scaffold 1, 2, 5, 7 and 8 (**Figure 2**).

### Gene structure and motifs of CcRRs

**Figure 3:**
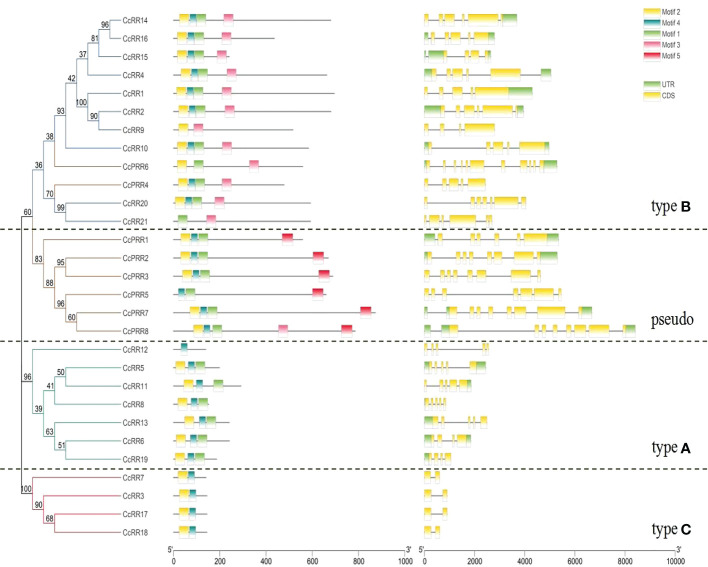
Phylogenetic relationship, conserved domains and exon-intron structures of CcRRs. Left: A phylogenetic tree constructed with the N-J method with MEGA 7. The proteins can be divided into four types, which are indicated with different colored lines (Green: type A; Blue: type B; Red: type C; Yellow: pseudo). Middle: Distribution of conserved domains of CcRRs. The relative positions of each domain are shown by colored bars (Motif 1, 2 and 4: D-D-K domain; Motif 3: MYB-binding domain; Motif 5: CCT domain). Right: The exons, introns and untranslated regions (UTRs) are represented by yellow rectangles, black lines and green rectangles, respectively.

The diversity of exon-intron structure and motifs may reveal the evolution of gene family (Wang et al., 2017; Sun et al., 2022). Intron-exon analysis of *CcRR* genes (**Figure 3**) indicated that the *CcRRs* classified into type A and B possessed 3 to 7 intronic regions, while there was only one intron in the type C *CcRRs*. The numbers of introns for *CcPRRs* varied more, ranging from 5 to 10. Five potential conserved motifs were identified from 29 CcRRs by online MEME program. The details of each motif were shown in **Supplementary Figure 2**. As shown in **Figure 3**, motifs 1, 2 and 4 encoding the D-D-K domain presented in most of CcRRs, but the sequences of D-D-K domain in some of CcRRs were incomplete. CcRR12 of type A, CcRR9 and CcRR21 of type B, CcPRR5 and CcPRR6 of pseudo RRs, and all type C CcRRs were missing part of the D-D-K domain sequence. All the type B CcRRs and CcPRR8 also possessed motif 3, which was identified as a conserved MYB-binding domain. Motif 5 was deemed as a conserved CCT motif and it existed in almost all CcPRRs except CcPRR4 and CcPRR6. These results suggested that the four types of CcRRs may play different roles in citrus.

### Prediction of *cis*-element in promoters of CcRR genes

**Figure 4:**
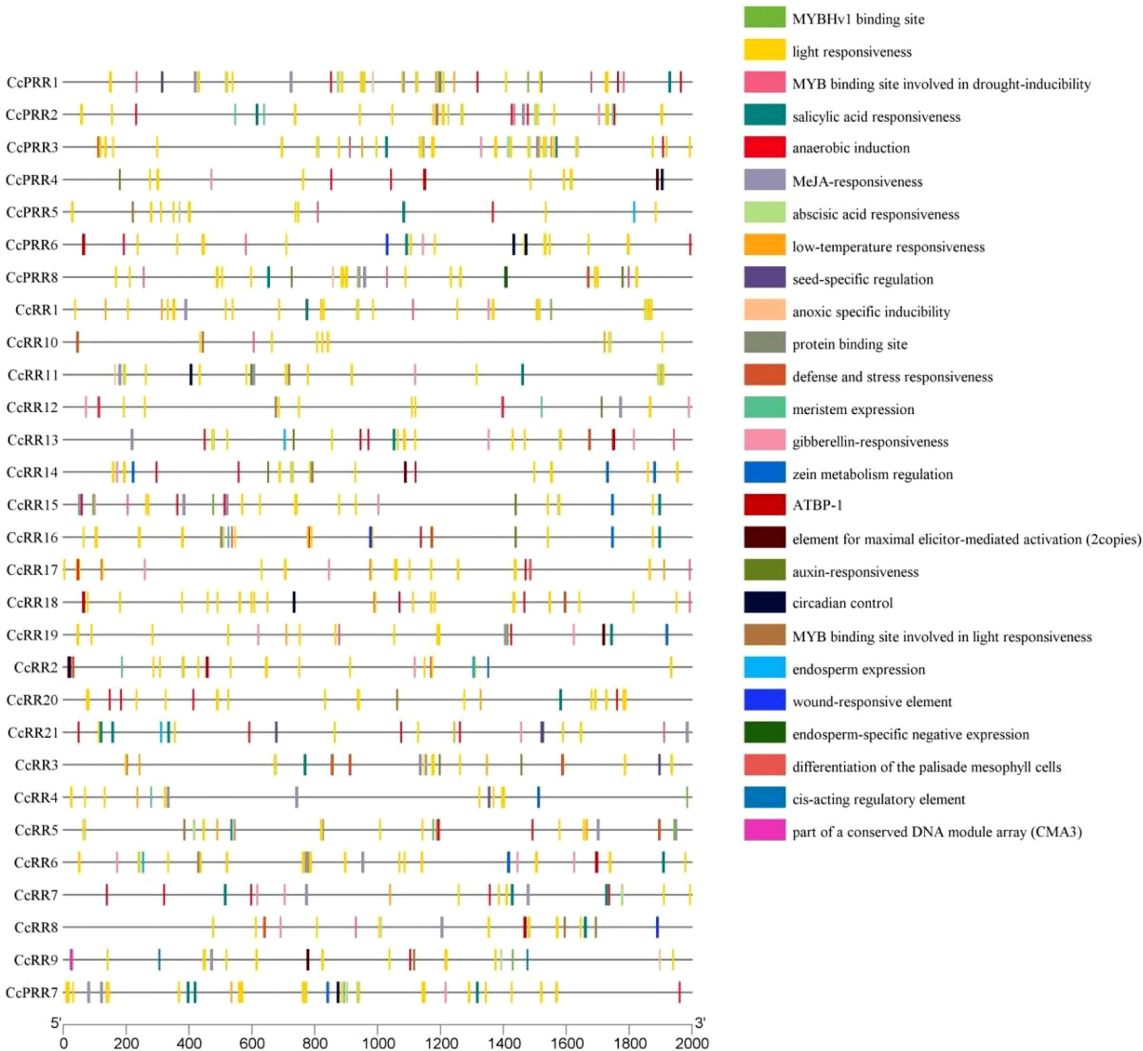
Predicted *cis*-acting elements in the promoter regions (2000 bp upstream of ATG start code) of *CcRRs*.

*Cis*-element in promoter region of the gene plays an important role in the regulation of gene transcription and usually combines with TFs to determine the level of gene expression (Wang et al., 2017; Dai et al., 2022). Results of *cis*-element prediction showed that the promoters of *CcRR* family members contain a large number of light responsiveness elements (**Figure 4**). Drought-induced, low-temperature response, defense and stress response elements were also found in promoter regions of *CcRRs*. In addition, there are many hormone response elements, such as abscisic acid, auxin, methyl jasmonate, salicylic acid, gibberellin response elements and zein metabolism regulation element. Some specific *cis*-acting elements were also identified, such as anaerobic induction, meristem development, and endosperm expression elements. The analysis of the predicted *cis*-elements in the promoter indicated that the transcriptional regulation of *CcRR* gene family members was probably related to the processes of growth and development of citrus plants, and the responses to various stresses.

### Tissue-specific expression profiles of *CcRR* genes

**Figure 5:**
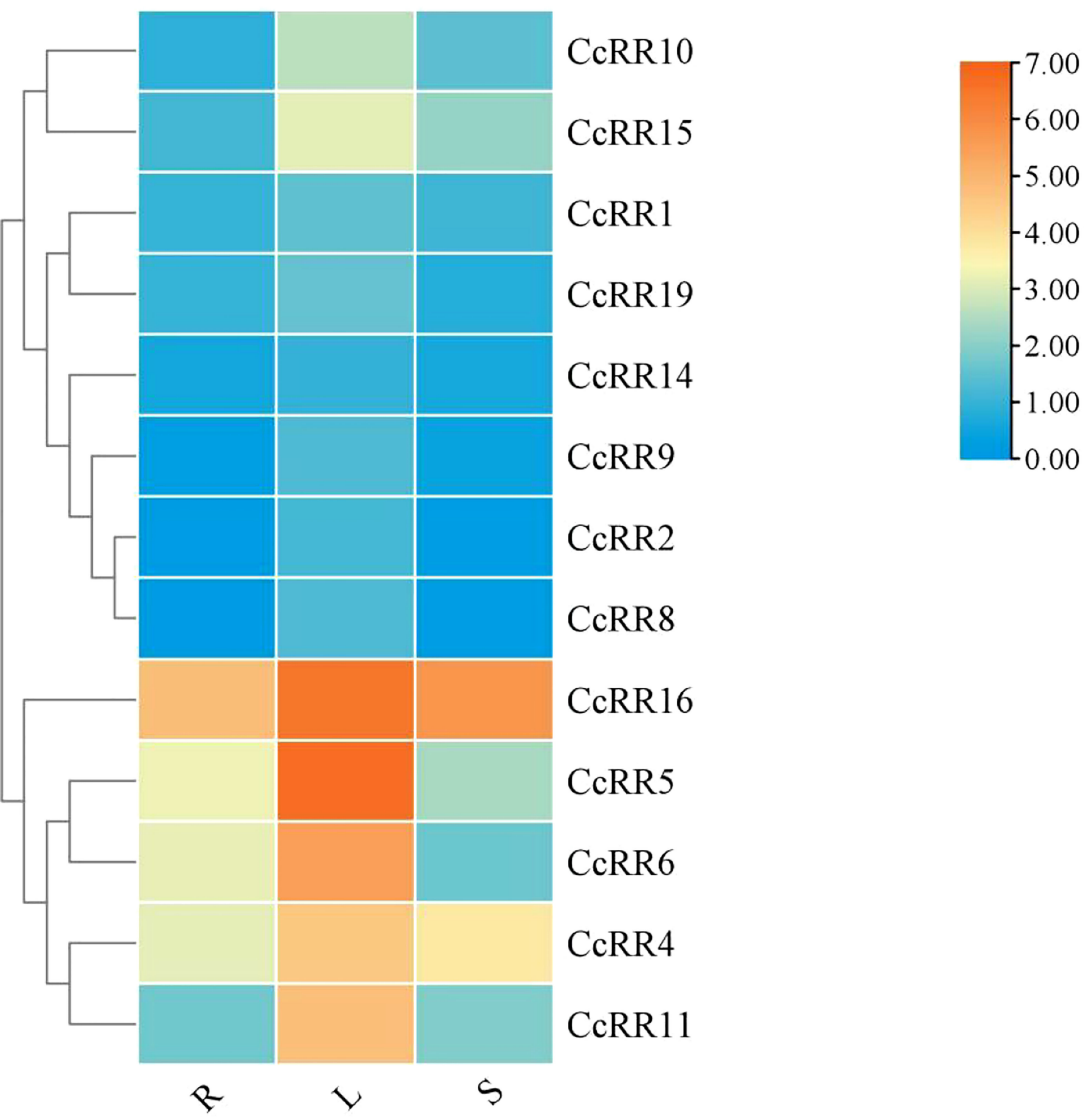
Heat-map of the expression profiles of *CcRR* genes in different tissues. R: root; L: leaf; S: stem. The vertical color scale shown to the right of the image represents log_2_ expression values.

Levels of gene expression in the roots, stems and leaves were analyzed by qRT-PCR to characterize the expression of type A and type B *CcRR* genes in different tissues of citrus (**Figure 5**). *CcRR12, CcRR13, CcRR20* and *CcRR21* expressed at very low level in roots, suggesting that they are not active in this tissue, therefore, they were excluded from further work of this study. The remaining 13 *CcRR* genes showed various expression patterns in different tissues, and most of them had the highest expression in leaves, such as *CcRR4-6*, *CcRR10*, *CcRR11*, *CcRR15* and *CcRR16*. Among them, *CcRR4*, *CcRR5*, *CcRR6* and *CcRR16* also highly expressed in roots.

### Expression of *CcRR* genes in response to phytohormones and abiotic stresses

**Figure 6:**
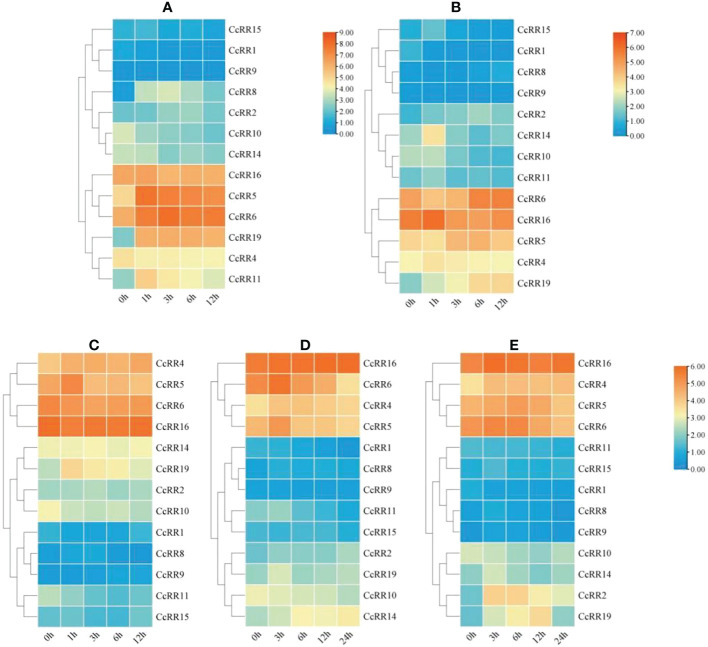
Heat-map of the expression pattern of *CcRR* genes in roots response to the treatments of hormone and abiotic stresses. **(A)** 6-BA treatment; **(B)** ABA treatment; **(C)** PEG treatment; **(D)** Cold treatment; **(E)** NaCl treatment. The figures **(C-E)** use the same ruler. The 0, 1, 3, 6, 12 and 24 h labels indicate the time after treatment. The vertical color scales shown to the right of the image represent log_2_ expression values.

Phytohormone and abiotic stress treatments could induce the expression of *CcRR* genes. Analysis of *RR* genes’ expression under hormone treatments and abiotic stresses showed that the transcriptional levels of *CcRR4*, *CcRR5*, *CcRR6*, *CcRR16* were high and changed significantly under the conditions of PEG, NaCl, cold, ABA or 6-BA treatment, indicating that those *CcRRs* dynamically responded to the multiple stresses (**Figure 6**).

Under 6-BA treatment (**Figure 6A**), the expressions of all the type A *CcRRs* (*CcRR5*, *CcRR6*, *CcRR8*, *CcRR11*, *CcRR19*) and some of type B *CcRRs* (*CcRR2*, *CcRR15*, *CcRR16*) were up-regulated at the early stage of the treatment and then declined, while the expressions of *CcRR1*, *CcRR4*, *CcRR9*, *CcRR10* and *CcRR14* were generally suppressed by the treatment. For ABA treatment (**Figure 6B**), the expression patterns of seven *CcRRs* (*CcRR2*, *CcRR4*, *CcRR5*, *CcRR11*, *CcRR14*, *CcRR15*, *CcRR16*) were basically the same. Their expressions up-regulated at the beginning of treatment and then declined, while the expression of *CcRR19* was increasing throughout the treatment. However, *CcRR1* and *CcRR10* showed the opposite trend of decreased expression, while the expressions of *CcRR6*, *CcRR8* and *CcRR9* were only down-regulated at the early stage, but increased afterward.

Under PEG treatment (**Figure 6C**), the expressions of four genes (*CcRR2*, *CcRR5*, *CcRR8* and *CcRR19*) were all up-regulated at 1h or 3 h after treatment, but down-regulated from 3h or 6h after treatment, while the expressions of *CcRR4* and *CcRR9* were up-regulated nearly in the whole period of treatment. On the opposite, the expression of *CcRR10* was gradually decreased during the treatment, while the expressions of five genes (*CcRR1*, *CcRR6*, *CcRR11*, *CcRR15*, *CcRR16*) were down-regulated at the early stage of treatment and then recovered at late stage. Under low temperature condition (**Figure 6D**), the expressions of *CcRR2*, *CcRR14* and *CcRR16* increased in the entire period of treatment, while the expression level of type A *CcRRs* (*CcRR5*, *CcRR6*, *CcRR8*, *CcRR11*, *CcRR19*) and *CcRR4* showed a trend of increasing first and then decreasing. However, the expressions of *CcRR1* and *CcRR10* were consistently decreasing, while the expressions of *CcRR9* and *CcRR15* were down-regulated at the early stage of treatment and then slight increased. For NaCl treatment (**Figure 6E**), the similar expression patterns were observed in the most of genes (*CcRR2*, *CcRR4*, *CcRR5*, *CcRR6*, *CcRR8*, *CcRR9*, *CcRR14*, *CcRR15*, *CcRR16* and *CcRR19*). Their expressions were also up-regulated at the beginning of treatment and then declined at varying degree. The expressions of *CcRR1* and *CcRR11* declined during the whole period of treatment, while the expression of *CcRR10* was down-regulated at the early stage of treatment and then up-expressed. The results of above observation indicated that most of *CcRR* genes involved in the processes responding to the various abiotic stresses and exogenous hormone treatments.

### Expression of *CcRR* genes in different zones of root and citrus varieties

**Figure 7:**
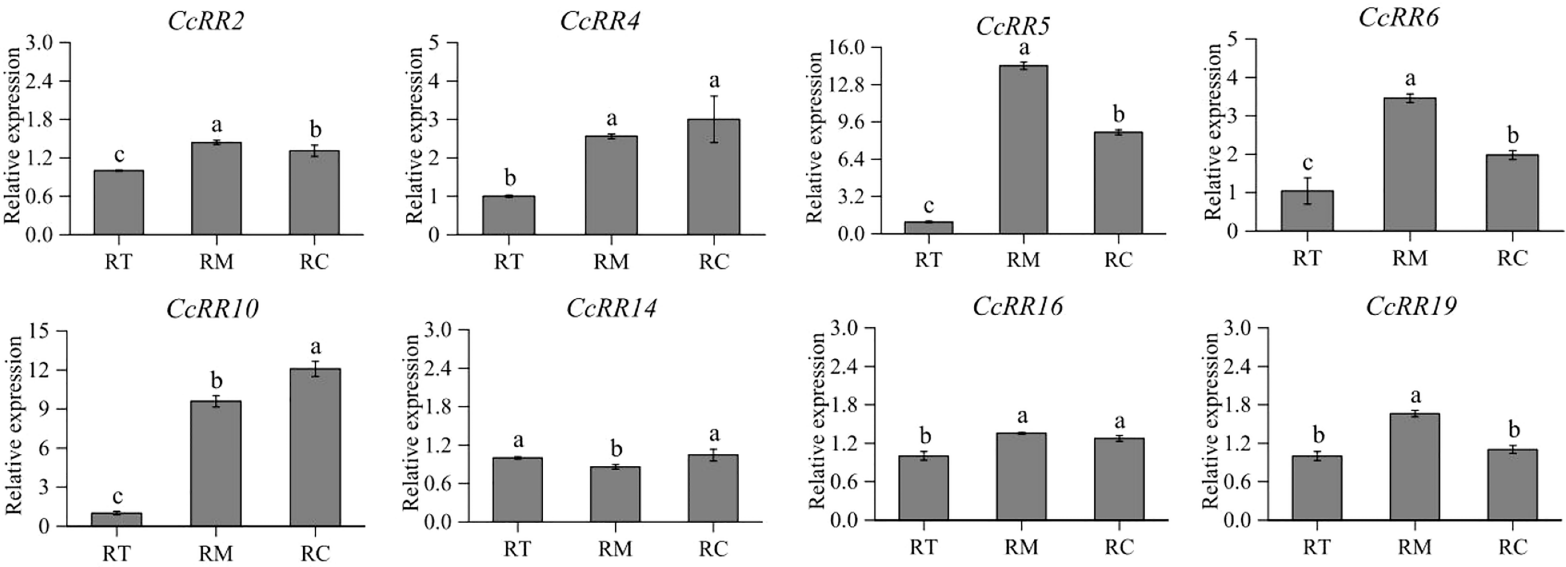
The relative expression of *CcRR* genes in different zones of citrus root. RT: the meristematic/elongation zone; RM: the root elongation/differentiation and lateral root initiation zone; RC: lateral root growth zone. The values are means ± SD of three independent biological replicates for qRT-PCR.

**Figure 8:**
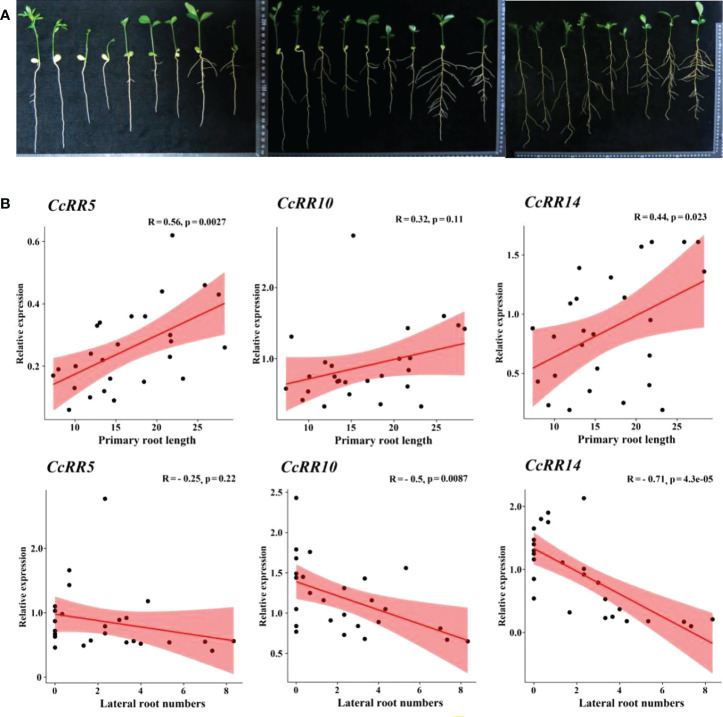
Root indexes and the correlation analysis of *CcRR5*, *10* and *14* at different stages. **(A)** Citrus root at 25, 50 and 75 days after sowning. From left to right are *Poncirus trifoliata* (Pt030, Pt038, Pt034, Pt026), *Citrus wilsonii* Tanaka ‘Zhique’, *C. reticulata* ‘Zhuju’, *C. reshni* Hort. Ex Tan ‘Cleopetra’, *C. limonia* Pasquale ‘Volkamer’, *C. limonia* Osbeck ‘Canton Lemon’, respectively; **(B)** Relationship between root indexes and their expressions.

The eight *CcRRs* highly expressed in roots, also drastically respond to the abiotic stresses and exogenous phytohormone treatments in above study were selected to explore their functions involved in root development with Volkamer seedlings. The levels of gene expression were investigated in RT, RM, and RC zones of the roots as shown in **Figure 7**. *CcRR2*, *CcRR5*, *CcRR6* and *CcRR19* predominately expressed in RM, among which the expression of *CcRR5* was the most prominent. The expressions of *CcRR4*, *CcRR16*, especially *CcRR10*, were higher in RC than that in RT. However, expression of *CcRR14* was significantly higher in both RT and RC than in RM. The results suggested that those *CcRRs* probably play different roles in root morphogenesis. Therefore, the three *CcRRs* (*CcRR5*, *CcRR10* and *CcRR14*) that distinguishingly expressed in different zones of root were further investigated to determine their roles in root morphogenesis of 9 citrus rootstocks, which varied in the length of primary roots and the numbers of lateral roots. Results of analysis indicated that the expression of *CcRR5* in RT zone was significantly correlated to the length of primary root, while the expression of *CcRR10* in RC zone was significantly correlated to the number of lateral roots, and the expressions of *CcRR14* were significantly correlated to the two root indexes (**Figure 8**; **Supplementary Table 2**).

## Discussion

Transcription factor *RRs* play crucial roles in the cytokinin signaling circuitry, due to their participation in multiple biological functions such as DNA-binding transcriptional regulation (Imamura et al., 1999; Sakai et al., 2001; Zeng et al., 2017). Members of the *RR* gene family have been identified in several woody plants, such as apple, pear, peach and poplar, and found to be involved in fruit development, adventitious root formation and primary root elongation (Singh and Kumar, 2012; Li et al., 2017; Ni et al., 2017; Zeng et al., 2017). However, in citrus, knowledge of *RRs* is very limited and the identification of *CcRR* gene family have not been reported. In this study, 29 RR members were bio-informatically identified from citrus genome. Phylogenetic analysis with *Arabidopsis* and poplar homologs classified those CcRRs into four types distinguished by their representative domains as reported in other species (Singh and Kumar, 2012; Li et al., 2017). Interestingly, each member of the CcRR family contains a D-D-K domain, suggesting that CcRRs may share a highly conserved function. However, the specific motifs and gene structures presented in different types of *CcRR* genes implied a diversified and physiological function of *CcRRs*.

Previous studies indicated that the expressions of the members of *RR* gene family were tissue specific in relating to their function (Ferreira and Kieber, 2005; Huang et al., 2015; Li et al., 2015). In the four types of *RRs*, type A and type B *RRs* play vital roles in aspects of plant growth and development (Jain et al., 2006; Bertheau et al., 2015). Type B *ARRs* can mediate lateral root formation under cold stress (Jeon et al., 2016). Type A and type B *ARRs* were reported to be involved in primary root growth, and type-B *ARRs* negatively modulate primary root growth *via AUX-1* mediated auxin translocation (Sharma et al., 2021). Therefore, the type A and type B *CcRRs* were targeted in this study. The expression patterns of type A and type B *CcRRs* were explored in root, stem and leaf. Among them, four genes (*CcRR4*, *CcRR5*, *CcRR6* and *CcRR16*) were highly expressed in roots and leaves. *CcRR12, CcRR13, CcRR20* and *CcRR21* were excluded from further work of this study because of their low activities in the root. Promoters of *CcRRs* contained the certain elements related to hormone responses and abiotic stresses, indicating their function may related to the hormone signaling and abiotic stress response same as in other plants (Jain et al., 2006; Ramírez-Carvajal et al., 2008; Tsai et al., 2012; Yang et al., 2016). This was proved by the results that 8 type A and type B *CcRRs* drastically responded to the treatments of exogenous hormones and abiotic stresses, partially as reported by Huang et al. (2018) in *Arabidopsis*. It was reported that root system architecture, including root growth and lateral root branching, was closely related to abiotic stresses resistance (Bielach et al., 2012). Functional analysis of homologous genes indicated that *CcRR14* and *CcRR16* might play roles in abiotic stress resistance in the root of citrus as they were grouped into the same clade with *ARR1*, *ARR2* and *PtRR13*. In this group, *ARR1* plays leading roles in many stress response processes, such as cold stress, salinity and ABA induction (Zhu et al., 2015; Li et al., 2015; Jeon et al., 2016; Yan et al., 2021), and *PtRR13* is a negative regulator of adventitious root development in *Populus* (Ramírez-Carvajal et al., 2009). In addition, *ARR1* and *ARR2* are response factors involved in primary root and lateral root meristem initiation, pathogen resistance, and leaf senescence (Hwang et al., 2012).

The specific expression of *RR* genes may relate to their function in root system architecture (Mason et al., 2004; Ferreira and Kieber, 2005; Garay-Arroyo et al., 2012). In this study, the tissue specific expression of *CcRR5*, *CcRR10* and *CcRR14* were significantly correlated to the characteristic indexes of root (primary root length and lateral root numbers) of nine citrus rootstocks. *CcRR5*, a type A *RR* gene highly expressed in RM, which is the elongation/differentiation and lateral root initiation zone of the root. Four *RRs* (*ARR5/6/7/15*) from *Arabidopsis* and one *RR* (*PtRR10*) from *Populus* were classified into the same subfamily with *CcRR5*. *PtRR10* is an ortholog of *ARR5* (Ramírez-Carvajal et al., 2008; Immanen et al., 2013). Those *ARRs* are as negative regulators of cytokinin signaling, participating in a negative feedback loop to reduce the sensitivity to cytokinin (Kiba et al., 2003; To et al., 2004; Leibfried et al., 2005; To et al., 2007). Cytokinin plays an inhibitory role on primary root growth arising from effects on cell division in the root meristem and on cell expansion in the root elongation zone (Kieber and Schaller, 2014). Therefore, it could be speculated that *CcRR5* should be involved in regulating root growth in citrus. *CcRR14* was classified into the same group as *ARR1*, which was reported to activate the expression of auxin signal inhibitor *SHY2* to regulate root meristem size in *Arabidopsis* (Li et al., 2020). Its expression significantly correlated to the length of primary root in nine citrus rootstocks, suggesting that it would be related to root elongation. Bielach et al. (2012) reported that the mutant of *arr1 arr11* in *Arabidopsis* (orthologues of *CcRR14* and *CcRR10*, respectively) enhanced cytokinin activity in pericycle cells, preventing lateral root initiation in close proximity to existing lateral root primordia. *CcRR10* predominantly expressed in the RC of root in citrus. The expressions of *CcRR10* and *CcRR14* were significantly correlated to the number of lateral roots. This clearly suggested that they are probably involved in the development of lateral roots. Results of this study indicated that *CcRR5*, *CcRR10* and *CcRR14* may play important roles in the process of root morphogenesis.

## Conclusions

Twenty-nine RR family members were identified from citrus genome and classified into four types based on their amino acid sequences and conserved domains. Expressions of type A and type B *CcRRs* varied in different tissues of plant and highly responded to phytohormones and abiotic stresses in the root. Three *CcRRs* (*CcRR5*, *CcRR10* and *CcRR14*) were identified to express at high levels in different zones of root, and their expression significantly correlated to the root characteristic indexes in nine citrus rootstocks. Functional analysis of their orthologs in *Arabidopsis* suggested that they may participate in regulating root morphogenesis.

## Data availability statement

The datasets presented in this study can be found in online repositories. The names of the repositories can be found in the article/**Supplementary Material**.

## Author contributions

MZ, SZ and XZ conceived and designed the experiments. MZ, FW, XW, JF and QY carried out the experiments. MZ, FW, SZ and XZ contributed to the data analysis. MZ, SZ and XZ contributed to paper writing and paper revising. All authors contributed to the article and approved the submitted version.

## Funding

This work was supported by the National Key Research and Development Program of China (2018YFD1000101) and China Agriculture Research System (CARS-Citrus) and the “Double World-classes” Development Plan of Southwest University.

## Conflict of interest

The authors declare that the research was conducted in the absence of any commercial or financial relationships that could be construed as a potential conflict of interest.

## Publisher’s note

All claims expressed in this article are solely those of the authors and do not necessarily represent those of their affiliated organizations, or those of the publisher, the editors and the reviewers. Any product that may be evaluated in this article, or claim that may be made by its manufacturer, is not guaranteed or endorsed by the publisher.
