# Supplementary material for: Mining key genes related to root morphogenesis through genome-wide identification and expression analysis of RR gene family in citrus
Source: Front Plant Sci. 2022 Nov 22;13:1068961. doi: 10.3389/fpls.2022.1068961 (PMC9725114; doi:10.3389/fpls.2022.1068961)
Supplement: Supplementary file 1 [file Image_1.pdf]

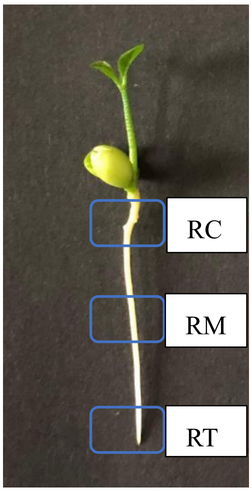

Figure S1 Root zones of Volkamer seedling. RT: the meristematic/elongation zone; RM: the root elongation/differentiation and lateral root initiation zone; RC: lateral root growth.
