# Supplementary figures and images for: Mining key genes related to root morphogenesis through genome-wide identification and expression analysis of RR gene family in citrus
Source: Front Plant Sci. 2022 Nov 22;13:1068961. doi: 10.3389/fpls.2022.1068961 (PMC9725114; doi:10.3389/fpls.2022.1068961)

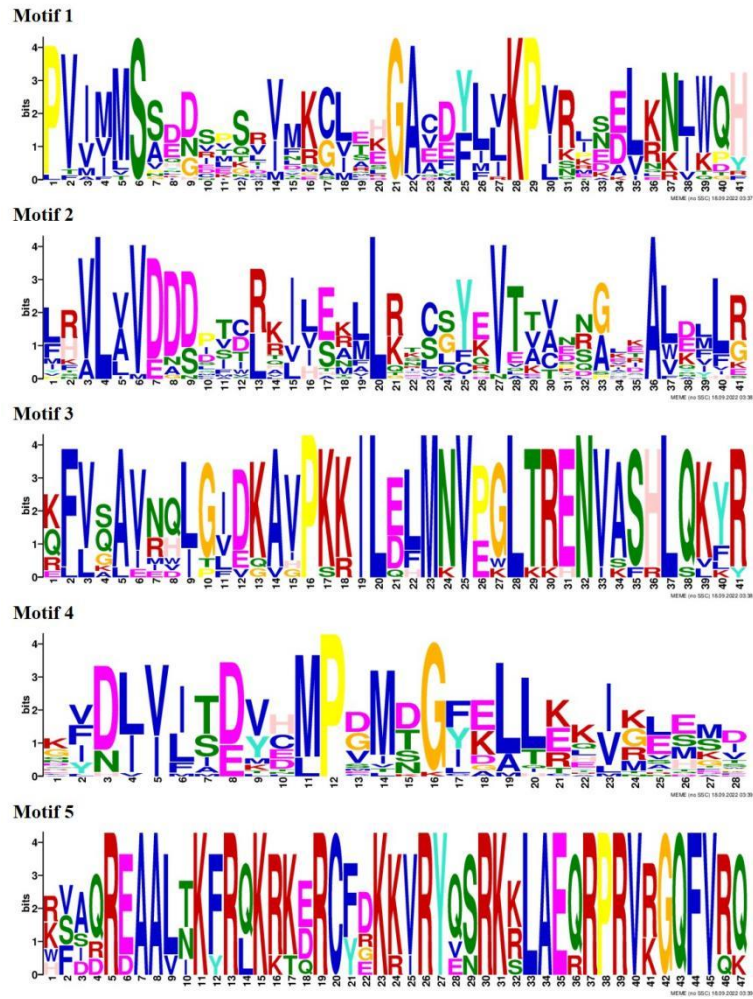

Figure S2 Conserved motifs of CcRRs identified by the online MEME program.

Supplement: Supplementary file 2 [file Image_2.pdf]
