# Supplementary material for: Mining key genes related to root morphogenesis through genome-wide identification and expression analysis of RR gene family in citrus
Source: Front Plant Sci. 2022 Nov 22;13:1068961. doi: 10.3389/fpls.2022.1068961 (PMC9725114; doi:10.3389/fpls.2022.1068961)
Supplement: Supplementary file 3 [file Table_1.doc]

Table S1 Primers of *CcRRs* used for qRT-PCR

| Genes | Gene | F | R |
| --- | --- | --- | --- |
| *CcRR1* | *Ciclev10007639m* | CATGAGTTCAATGGTGAAAGAGGT | GTGCGGAACCACAACAATAGAC |
| *CcRR2* | *Ciclev10007648m* | TTACGTATCGGTGCACTGGA | CAAACATGTTGCCCGGTACA |
| *CcRR4* | *Ciclev10014525m* | AGAATAGTGCAGTCCTCGGG | TAGAGGCATCGACGAAGCAT |
| *CcRR5* | *Ciclev10022334m* | CATGCCTGGGATGACTGGAT | ATTCCTCTGCCCCTTCTTCC |
| *CcRR6* | *Ciclev10021937m* | TTGAAGCCAGTCCGATTGTC | TTGTTGCTGTGGTGGTGATG |
| *CcRR8* | *Ciclev10032983m* | ATGCCAGGAATGACGGGATA | GCATGAACATTTGAGCCCCT |
| *CcRR9* | *Ciclev10033605m* | CAGGCATGATCGGCAATGAA | CTCGAGCTAAAGGGACCCAT |
| *CcRR10* | *Ciclev10031052m* | TGGACCCTTGCTTACTGGAG | TGAAGAAGCATCCCCACAGT |
| *CcRR11* | *Ciclev10032281m* | ATCTTGAGCAGGAGTCATCTGC | CTGCTCCGTCCTCTAAACATCT |
| *CcRR12* | *Ciclev10003634m* | TGACCGGATGCGAGTTGATT | ATACTGAATCCTTGCCGGCT |
| *CcRR13* | *Ciclev10002312m* | TCGTGGTCATGTCTTCCGAA | TCGCTGTCTTCATTGTTGCC |
| *CcRR14* | *Ciclev10004470m* | GGTCAGCATCTCAATAGCCTTC | CCATCCTGCTGCTGTTTCAG |
| *CcRR15* | *Ciclev10005747m* | TGATGTCTGTGGATGGTTGCAC | TGAGCCACATGCTGCCATATG |
| *CcRR16* | *Ciclev10004999m* | CGGCAACCAAAACCATCTGA | ACGCGGCTTCTTTAAAGTGG |
| *CcRR19* | *Ciclev10005987m* | CACGTTCTTGCTGTTGACGA | TCCAAAGCCTTGTTACCAGAA |
| *CcRR20* | *Ciclev10006586m* | CAGCTTTGGTTCCATTGGCT | AAATGTGGTGCCAGGGTTTG |
| *CcRR21*  *CcActin* | *Ciclev10006834m*  *Ciclev10025866m* | AGGTGGCTTGGACTGATTCA  CCGACCGTATGAGCAAGGAAA | GCTAGCCACATTTTCCCTCG  TTCCTGTGGACAATGGATGGA |
