# Supplementary material for: Mining key genes related to root morphogenesis through genome-wide identification and expression analysis of RR gene family in citrus
Source: Front Plant Sci. 2022 Nov 22;13:1068961. doi: 10.3389/fpls.2022.1068961 (PMC9725114; doi:10.3389/fpls.2022.1068961)
Supplement: Supplementary file 4 [file Table_2.doc]

Table S2 Root indexes and the relative expression of *CcRR5*, *10*, and *14* in nine citrus rootstock varieties

| Variety | Primary root length (cm) | Relative expression of *CcRR14*-RT | Relative expression of *CcRR10*-RT | Relative expression of *CcRR5*-RT | Lateral root numbers | Relative expression of *CcRR14*-RC | Relative expression of *CcRR10*-RC | Relative expression of *CcRR5*-RC | |
| --- | --- | --- | --- | --- | --- | --- | --- | --- | --- |
| Pt034-25d | 9.93 | 0.81 | 0.54 | 0.13 | 0 | 1.3 | 1.44 | 0.63 |  |
| Pt034-50d | 16.9 | 1.31 | 0.69 | 0.36 | 2.33 | 0.92 | 0.98 | 0.79 |  |
| Pt034-75d | 27.57 | 1.61 | 1.47 | 0.43 | 0.67 | 1.75 | 1.25 | 1.43 |  |
| Pt026-25d | 7.33 | 0.88 | 0.58 | 0.17 | 0 | 1.16 | 1.49 | 0.67 |  |
| Pt026-50d | 13.03 | 1.39 | 0.75 | 0.34 | 0 | 0.85 | 0.84 | 0.72 |  |
| Pt026-75d | 21.9 | 1.61 | 1.01 | 0.62 | 0.33 | 1.8 | 1.45 | 0.98 |  |
| Pt038-25d | 13.57 | 0.86 | 0.69 | 0.12 | 0 | 1.65 | 1.79 | 0.87 |  |
| Pt038-50d | 20.63 | 1.57 | 1 | 0.44 | 0 | 1.4 | 1.05 | 0.87 |  |
| Pt038-75d | 25.87 | 1.61 | 1.6 | 0.46 | 0.67 | 1.9 | 1.76 | 1.66 |  |
| Pt030-25d | 14.77 | 0.83 | 0.5 | 0.09 | 0 | 1.47 | 1.68 | 1.03 |  |
| Pt030-50d | 21.73 | 0.95 | 0.84 | 0.28 | 0 | 1.25 | 0.77 | 1.1 |  |
| Pt030-75d | 28.3 | 1.36 | 1.42 | 0.26 | 2.33 | 2.13 | 1.31 | 2.77 |  |
| Canton-25d | 9.27 | 0.23 | 0.42 | 0.06 | 3.67 | 0.25 | 1.16 | 0.56 |  |
| Canton-50d | 14.3 | 0.35 | 0.67 | 0.16 | 4.33 | 0.18 | 1.05 | 1.18 |  |
| Canton-75d | 21.6 | 0.4 | 0.61 | 0.23 | 8.33 | 0.21 | 0.65 | 0.56 |  |
| Volkamer-25d | 11.83 | 0.19 | 0.33 | 0.1 | 5.33 | 0.18 | 1.56 | 0.54 |  |
| Volkamer-50d | 18.43 | 0.25 | 0.36 | 0.15 | 7 | 0.17 | 0.81 | 0.55 |  |
| Volkamer-75d | 23.2 | 0.19 | 0.33 | 0.16 | 7.33 | 0.1 | 0.67 | 0.41 |  |
| Cleopetra-25d | 10.07 | 0.48 | 0.75 | 0.2 | 0 | 0.54 | 2.43 | 0.46 |  |
| Cleopetra-50d | 13.37 | 0.74 | 0.68 | 0.22 | 3.33 | 0.53 | 1.43 | 0.92 |  |
| Cleopetra-75d | 21.63 | 0.65 | 1.43 | 0.3 | 4 | 0.37 | 0.89 | 0.52 |  |
| Zhique-25d | 11.93 | 1.09 | 0.95 | 0.24 | 1.33 | 1.11 | 1.16 | 0.49 |  |
| Zhique-50d | 12.7 | 1.13 | 0.9 | 0.33 | 2.33 | 1.01 | 0.73 | 0.68 |  |
| Zhique-75d | 18.57 | 1.14 | 0.76 | 0.36 | 3 | 0.79 | 0.84 | 0.89 |  |
| Zhuju-25d | 7.97 | 0.43 | 1.31 | 0.19 | — | — | — | — |  |
| Zhuju-50d | — | — | — | — | 1.67 | 0.32 | 0.91 | 0.57 |  |
| Zhuju-75d | 15.23 | 0.54 | 2.73 | 0.27 | 3.33 | 0.23 | 0.68 | 0.54 |  |
